# Effect of the Organic Functional Group on the Grafting Ability of Trialkoxysilanes onto Graphene Oxide: A Combined NMR, XRD, and ESR Study

**DOI:** 10.3390/ma12233828

**Published:** 2019-11-21

**Authors:** Massimo Calovi, Emanuela Callone, Riccardo Ceccato, Flavio Deflorian, Stefano Rossi, Sandra Dirè

**Affiliations:** 1 Department of Industrial Engineering, University of Trento, 38123 Trento, Italy; 2 “Klaus Müller” Magnetic Resonance Laboratory, Department of Industrial Engineering, University of Trento, 38123 Trento, Italy

**Keywords:** graphene oxide, organosilanes, grafting, solid state NMR, XRD, ESR

## Abstract

The functional properties displayed by graphene oxide (GO)-polymer nanocomposites are strongly affected by the dispersion ability of GO sheets in the polymeric matrix, which can be largely improved by functionalization with organosilanes. The grafting to GO of organosilanes with the general formula RSi(OCH_3_)_3_ is generally explained by the condensation reactions of silanols with GO reactive groups. In this study, the influence of the organic group on the RSi(OCH_3_)_3_ grafting ability was analyzed in depth, taking into account the interactions of the R end chain group with GO oxidized groups. Model systems composed of commercial graphene oxide reacted with 3-aminopropyltrimethoxysilane (APTMS), 3-mercaptopropyltrimethoxysilane (MPTMS), and 3-methacryloxypropyltrimethoxysilane, (MaPTMS), respectively, were characterized by natural abundance ^13^C, ^15^N and ^29^Si solid state nuclear magnetic resonance (NMR), x-ray diffraction (XRD), and electron spin resonance (ESR). The silane organic tail significantly impacts the grafting, both in terms of the degree of functionalization and direct interaction with GO reactive sites. Both the NMR and XRD proved that this is particularly relevant for APTMS and to a lower extent for MPTMS. Moreover, the epoxy functional groups on the GO sheets appeared to be the preferential anchoring sites for the silane condensation reaction. The characterization approach was applied to the GO samples prepared by the nitric acid etching of graphene and functionalized with the same organosilanes, which were used as a filler in acrylic coatings obtained by cataphoresis, making it possible to correlate the structural properties and the corrosion protection ability of the layers.

## 1. Introduction

Recently, large interest has been devoted to the use of graphene as a nanofiller in polymeric matrices thanks to its peculiar features [1,2]. To ensure the effective improvement of polymer properties by graphene addition, additives are required in order to obtain a homogeneous distribution within the matrix. In this regard, graphene flakes are usually oxidized to obtain graphene oxide (GO) [3,4], which is subsequently functionalized to increase chemical affinity with the polymer matrix [5].

Among the different functionalizing agents, organoalkoxysilanes have been widely used with success, exploiting both their grafting ability [6,7] and the possibility to ad hoc select the end chain organic functionality. Organoalkoxysilanes with the general formula RSi(OR’)_3_ are characterized by two different functional groups: -OR’ are hydrolyzable groups forming reactive silanols suitable for condensation reactions, while R is a non-hydrolyzable organic group that imparts the desired features [8]. Generally, organoalkoxysilane grafting to GO is considered to take place by condensation reactions among silanols and the functional groups present on the basal plane of the GO flakes, producing an increase in the interplanar distance of the lamellae that depends on the silane organic chain, with the consequence of improving the dispersion in a polymer matrix [9].

The recent literature reports several studies on GO functionalization with silanes for a wide range of applications. For example, through the reaction of vinyltrimethoxysilane (VTMS) and GO in an aqueous medium, Abass et al. obtained VTMS-reduced graphene oxide (rGO) nanospheres and pointed out the role of silane in both the successful exfoliation of rGO layers and the enhancement of the electrical conductivity [9]. With the final aim to prepare high-performance thermosetting resins, Xu et al. modified GO with aminopropyltrimethoxysilane (APTMS); after reduction with hydrazine, the thermal resistance of functionalized GO appeared improved as did its dispersion ability in organic solvent, allowing the use of APTMS-rGO as filler for polybenzoxazine resins [10]. Moreover, Chen et al. studied the APTMS-grafted GO as filler for polybenzoxazole (PBO) fibers and obtained a new hierarchical reinforcement. They proved that not only the surface roughness and wettability of the PBO fibers increased after grafting GO with APTMS, but the atomic oxygen erosion resistance of PBO fibers and its composites was also improved, making these materials suitable for aerospace applications [11].

In previous reports [12,13], we showed that the addition of silane-functionalized GO flakes to an acrylic cataphoretic bath allowed for an increase in the corrosion resistance properties of the composite coatings. GO was produced by nitric acid etching of commercial graphene, obtaining flakes with a low degree of oxidation suitable for preserving the electrical properties of the pristine material. The graphene oxide flakes were functionalized with trialkoxysilanes with R groups characterized by different end chain functions, obtaining different results both in terms of the dispersion of the lamellae into the polymer matrix and the properties of the protective layers. Interestingly, aside from the proof of successful grafting and good dispersion ability of silane-modified GO flakes in the cataphoretic bath, the results showed that not only the filler load but, above all, the choice of the silane, had a key role in obtaining the desired properties in the final coating.

In order to improve the design of composite coatings with functionalized GO, avoiding trial–error processes and reducing the production costs of the final product, a fundamental step is the understanding of the structure-properties relationships of the material. The different reactivity toward GO of the used organoalkoxysilanes pointed out that the organic functional group R plays a fundamental role in the grafting process, which needs to be clarified. Unfortunately, the obtained silanized GO flakes [12] presented a low degree of functionalization that was sufficient to increase the dispersion ability and compatibility with the acrylic resin, but was not suitable for a deep structural characterization of the materials. Therefore, a model GO sample with a high degree of oxidation (i.e., a high amount of functional groups available as anchoring sites for trialkoxysilanes) must be selected in order to exploit the information that can be obtained through the combination of different spectroscopic techniques. Accordingly, the commercial product Graphenea (Ga), was functionalized with the organoalkoxysilanes bearing different end chain groups, following the procedure adopted in Calovi et al. [13], and the samples were characterized by solid state nuclear magnetic resonance (NMR) analysis and x-ray powder diffraction (XRD) to study the degree of functionalization, the preferential anchoring sites, and the type of interaction between silane and GO. Moreover, electron spin resonance (ESR) was used to evaluate the type and amount of GO conductive defects in correlation with the different degree of functionalization obtainable with the three silanes.

## 2. Materials and Methods

3-aminopropyltrimethoxysilane (APTMS Merck KGaA, Darmstadt, Germany), 3-mercaptopropyltrimethoxysilane (MPTMS Merck KGaA, Darmstadt, Germany), and 3-methacryloxypropyltrimethoxysilane (MaPTMS, Merck KGaA, Darmstadt, Germany), nitric acid (Merck KGaA, Darmstadt, Germany), toluene (Merck KGaA, Darmstadt, Germany), ethanol (Merck KGaA, Darmstadt, Germany), and acetone (Merck KGaA, Darmstadt, Germany) were used as received. Graphene powder (G, COMETOX s.r.l. (Milan, Italy)) with an average diameter of 25 µm was provided by COMETOX s.r.l. The graphene oxide aqueous dispersion (0.4 wt.% concentration, Graphenea Inc., Cambridge, MA, USA) was supplied by Graphenea (Donostia, Gipuzkoa, Spain).

Graphene oxide powders (GO) were obtained by the reaction of G with nitric acid, according to [12]. GO powders were reacted in a 1:0.1 molar ratio with 3-aminopropyltrimethoxysilane, 3-mercaptopropyltrimethoxysilane, and 3-methacryloxypropyltrimethoxysilane [12], and the functionalized samples were labeled GO-N, GO-S, and GO-M, respectively.

**Scheme 1:**
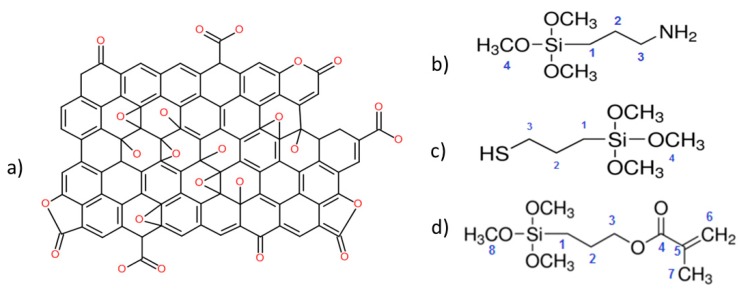
Molecular structure of (**a**) Graphenea, (**b**) 3-aminopropyltrimethoxysilane (APTMS), (**c**) 3-mercaptopropyltrimethoxysilane (MPTMS), and (**d**) 3-methacryloxypropyltrimethoxysilane (MaPTMS). The numbering of carbon atoms used for the peak assignment in the nuclear magnetic resonance (NMR) spectra is also shown.

The Ga sample was obtained by drying the graphene oxide aqueous dispersion at 60 °C for 24 h and grinding the solid residues with a mortar. Ga powders were then subjected to the functionalization process with APTMS, MPTMS, and MaPTMS in a 1:0.1 molar ratio under the same conditions employed for the GO powders. The functionalized samples were labeled Ga-N, Ga-S, and Ga-M, respectively. Scheme 1 shows the structures of both Ga and trialkoxysilanes used for the functionalization.

Solid state NMR analyses were carried out with a Bruker 400WB spectrometer (Bruker, Billerica, MA, US) operating at a proton frequency of 400.13 MHz. The magic angle spinning (MAS) NMR spectra were acquired with cross-polarization (CP) and noise dephasing single pulse (SP) pulse sequences under the following conditions: ^13^C frequency, 100.48 MHz; π/2 pulse 3.5 μs; decoupling length 5.9 µs; 7 k scans and recycle delay 15 s. For CP: recycle delay 5 s and 20 k scans; contact time 0.5 ms; proton decoupled pulse π/4; pulse 2.5 μs; recycle delay 10; and 128 scans. ^29^Si frequency, 79.49 MHz; π /2 pulse 4.1 μs; contact time 5 ms; decoupling length 5.9 µs; 10 k scans; and recycle delay 10 s. The samples, diluted with KBr in order to avoid skin depth effect (RF penetration) and probe tuning problems [14], were packed in 4 mm zirconia rotor and spun at 10 kHz under air flow. ^15^N frequency 40.54 MHz; π /2 pulse 2.2 μs; contact time 2 ms; decoupling length 5.9 µs; 80 k scans; and recycle delay 3 s. Adamantane, Q_8_M_8_, and glycine were used as external secondary references. The silicon sites were labeled according to the usual T^n^ notation, where T represents the trifunctional SiCO_3_ unit and n (n = 0 ÷ 3) is the number of bridging oxygen atoms. The lineshape analysis was performed using Bruker TopSpin software and the fitting was considered acceptable with a confidence level of 90%.

The ESR (Bruker, Billerica, MA, US) spectra were acquired at room temperature with a Bruker EMX cw spectrometer equipped with a rectangular cavity working in the X band at 9.77 GHz microwave frequency with a modulation frequency of 100 kHz. The intensity of the signal was normalized with respect to the weight of the powder sample. The magnetic field and g-value were calibrated with the DPPH powder sample (diphenyl picrylhydrazyl free radical, g = 2.0036).

Powder x-ray diffraction spectra were collected by means of a Rigaku D-Max III-D powder diffractometer (Rigaku, Tokyo, Japan) using Cu-Kα radiation (λ = 0.154056 nm) and a graphite monochromator in the diffracted beam. A θ–2θ Bragg-Brentano configuration was adopted with the following scan conditions: scan range 3–80° (in 2θ); and a sampling interval and counting time of 0.05° and 5 s, respectively. Jade8^®^ software (MDI, Livermore, CA, USA) was used for the fitting procedure of the experimental peaks in order to evaluate the peak position and full width at half maximum (FWHM) values, after the background correction.

## 3. Results and Discussion

A requirement to properly characterize the interaction between functionalizing agents and graphene oxide is the availability of a valuable number of reactive sites on the sheets. Three organotrimethoxysilanes (H_3_CO)_3_Si–CH_2_–CH_2_–CH_2_–X were used with the objective of assessing the effect of the end chain group X (X = –NH_2_, –SH and –(OOC)–CH_2_=CH–CH_3_) on the reactivity toward graphene oxide functionalization and possibly elucidate the preferential organosilane-GO interactions. A commercial high oxidation degree graphene oxide (Ga) was employed for the combined spectroscopic study, aiming to possibly state a correlation with the properties imparted by different silane-functionalized graphene oxide powders to protective layers, as reported in our previous study [12].

**Figure 1:**
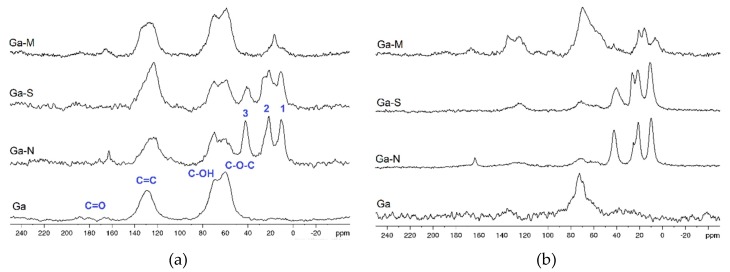
^13^C solid state NMR spectra of the samples Ga, Ga-N, Ga-S, and Ga-M. (**a**) Decoupled MAS and (**b**) CPMAS.

^13^C solid state NMR is suitable to explore the structural features of the graphene oxide flakes and the extent of silane grafting. Accordingly, both the ^13^C proton decoupled MAS (Figure 1a) and CPMAS (Figure 1b) experiments were run on pristine and functionalized Ga. The spectra resolution was good enough to point out the presence of different functional groups on the graphene layers.

The two main resonances in the Ga ^13^C MAS NMR spectrum (Figure 1a) belonged to sp^2^ aromatic carbons (about 130 ppm) and alcoholic and epoxide groups (about 70 and 59 ppm, respectively), respectively. Two weak and broad bands were also detectable in the range of 200 ÷ 160 ppm, which can be attributed to small amounts of ketones and edge carboxyls [15]. The main identified resonances in the spectrum of Ga are summarized in Table 1 [16].

The amount of the different functional groups was evaluated from the profile fitting analysis of the quantitative Ga spectrum (Table 1, Figure S1). With respect to a pure graphene sheet that should give rise to a single resonance at about 100–130 ppm, and taking into account all the identified carbon groups, the sp^2^ carbons were about 30% of the total, therefore assessing the high degree of oxidation of Ga sample.

Figure 1a also shows the spectra of the functionalized Ga samples. In the Ga-N and Ga-S spectra, the peaks of the α-, β-, and γ-methylene groups of the propyl chains (labeled 1, 2, and 3 in Figure 1a according to Scheme 1) of APTMS and MPTMS, respectively, are clearly detected in the range of 0–50 ppm. In contrast, unresolved broad resonances both in the methylene region and carbonyl range (167 ppm) characterize the Ga-M spectrum. Interestingly, in the carbonyl region of the Ga-N spectrum, a sharp peak was detected at 163.2 ppm, whose features appeared different with respect to those of the signals found in pristine Ga; its not negligible presence both in the MAS and CPMAS spectra (Figure 1a,b) of Ga-N deserves a more in-depth description, presented further on. In Ga-N and Ga-S, the aromatic resonances (150–100 ppm) showed an upfield shift of about 5 ppm and the signal lineshape changed as a consequence of the functionalization, particularly for Ga-S. Instead, the Ga-M sample did not show remarkable differences for the aromatic peak, maintaining the same lineshape with an upfield shift less than 2 ppm.

It is worth noting that in Ga-N and Ga-S, the silane functionalization led to a modification of the epoxy resonance, which appeared broader and reduced in intensity with respect to the one in pristine Ga. Conversely, the signal appeared almost unchanged in the Ga-M sample.

Table 2 reports, for both the pristine and functionalized Ga samples, the total peak area of the oxidized groups (C–OH, C–O–C, and C=O) were normalized considering the aromatic signal (in the range of 134–125 ppm). Considering the low number of carbonyl groups (Table 1), the Ga functionalization degree can be estimated by comparing the relative intensity of alcoholic and epoxy signals and those due to α-, β-, and γ-methylene carbons of the organosilanes. The most effective functionalization was found in Ga-N, whereas the degree of grafting was almost negligible in the case of Ga-M, as also confirmed by the Fourier transform infrared (FTIR) analysis (Figure S2).

The profile fitting analysis of the spectra permits the calculation of the amount of alcoholic and epoxide functions in the different samples to appreciate the different consumption of the Ga reactive sites with changing the employed silane in the grafting process. The COH/COC (epoxy) ratio (Table 2) suggests that the epoxide groups are the preferential anchoring sites in Ga-S and even more so in Ga-N, whereas the epoxide involvement appeared negligible in Ga-M. This indicates that the reactive organic groups linked to the silane propyl chain have a relevant influence on the grafting ability onto the graphene oxide surface, which probably overcomes the well-known electronic inductive effect on the condensation ability of the alkoxide group [17]. In particular, the preferential consumption of the epoxide functions in Ga-N suggests that the amine terminal group could play a role in the grafting, in addition to the expected anchoring obtained through methoxy group condensation. As a matter of fact, the epoxide opening should generate both C–OH and C–NH groups in the GO sheet, whose ^13^C signals should be at about 70 and 60 ppm, justifying the noticed increase in the C–OH peak. The direct interaction of the end sites is expected to be less relevant with the thiol group and negligible for the methacrylate one.

It must be mentioned that in the literature the –COOH group has been reported as the preferential anchoring site, leading to the possible formation of amide and thioester derivatives [18]. Unfortunately, the –COOH amount in our samples was very low and too broad to be evaluated quantitatively for a detailed discussion. Qualitatively, it can be seen that the –COOH related peaks were not anymore detectable in Ga-N (where a small peak at about 164 ppm suggests the formation of an amide group) and in the Ga-S spectra (no thioester signals at about 200 ppm are visible); in contrary, the –COOH broad resonances seemed unchanged in Ga-M, for which the overall grafting was quite poor.

To obtain more insight into the interactions between Ga and organosilanes, the ^13^C CPMAS spectra have been analyzed. The improved resolution achievable for sp^3^ carbons can be appreciated in particular from the signal to noise (S/N) ratio in the Ga-N and Ga-S spectra (Figure 1b).

By studying in detail the resonances of the methylene carbons of the APTMS propyl chain in the Ga-N spectrum, the following information were obtained: (i) the upfield position of Cα at about 10 ppm, and the absence of –OMe peaks indicate a good condensation degree; (ii) the Cβ peak splits in two resonances at 25.4 and 21.0 ppm, suggesting the presence of amino groups with different protonation degrees, since it is well known that the second carbon close to the terminal nitrogen is sensitive to its changes [19,20]; and (iii) the peak at 42 ppm belongs to Cγ and results were quite insensitive to the structural rearrangements.

The two main broad peaks observable in the spectra of functionalized samples (Figure 1b) belong to Ga. The one centered at 125 ppm is due to the aromatic carbons and the two peaks at 71.2 and 59.7 ppm were assigned to alcoholic and epoxide functions, which were also detected in the CPMAS spectrum of pristine Ga (Figure 1b). Moreover, as remarked above for the MAS spectrum, in the carbonyl region of Ga-N, a sharp resonance was detected at 163.2 ppm, which can be tentatively attributed to edge carboxyl carbons [15] or amide groups [21,22,23].

To shed light on the peculiarities shown by the Ga-N sample, and further investigate the interaction of the aminopropyl chains with the Ga sheets, a sample with Ga:APTMS = 1:1 molar ratio was prepared. Due to the high APTMS amount, it is likely that both the grafting process and the silane self-condensation reaction take place. Focusing on the Cβ resonance (^13^C CPMAS spectrum, Figure S3), the intensity of the downfield component (25 ppm) increased when compared to the same resonance in the Ga-N spectrum of Figure 1b, reaching roughly the 1:1 ratio with the upfield component (21 ppm). Moreover, the intensity of the signal at 163 ppm also increased with respect to the Ga-N spectrum.

These results prompted us to record a long ^15^N CPMAS experiment at nitrogen natural abundance.

**Figure 2:**
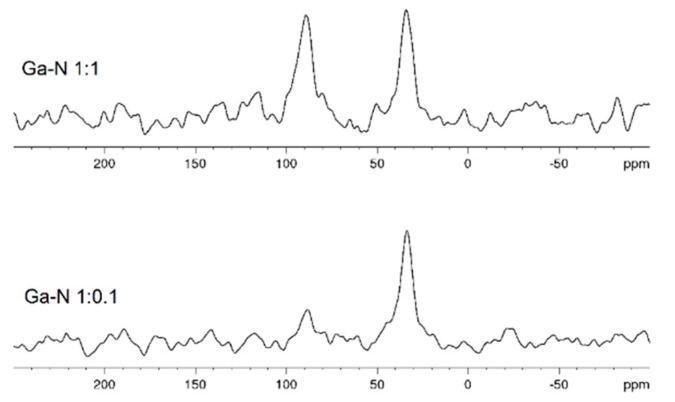
^15^N CPMAS NMR spectra of the sample Ga-N prepared with the Ga:APTMS ratio of 1:1 and 1:0.1, respectively.

Excitingly, the ^15^N spectra of both the Ga-N 1:01 and Ga 1:1 samples showed two resonances at 33 and 89 ppm (Figure 2), which can be attributed to the primary amine and amide functional groups, respectively [21,23].e The two peaks presented an intensity ratio 1:0.25 for the Ga-N 1:0.1 sample, reaching a ratio of 1:1 in the Ga with a higher APTMS load. This clearly proves that the unexpected carbon resonance at about 163 ppm, present only in the Ga-N spectra, can be attributed to the reaction between edge carboxylic and amino groups, probably belonging to two subsequent graphene layers. Accordingly, the possibility of APTMS anchoring graphene oxide layers both through Si–O–C and O=C–NH– bond formation explains its highest ability among the chosen silane series.

In the ^13^C CPMAS spectrum of Ga-S (Figure 1b), the peaks due to the methoxy groups were absent, representing a complete hydrolysis. The spectrum shows the Ga resonances, and the peaks due to the mercaptopropyl chain were found at 10.8 (Cα), 21.3 and 26.3 (Cβ), and 40.2 ppm (Cγ), respectively. Interestingly, in this case, the Cβ resonance appeared to split into two components and the occurrence of chain folding with different –SH interaction could be adduced. It is known that upon MPTMS condensation, the detection in the spectrum of two signals at about 11 (Cα) and 28 ppm (Cβ and Cγ) indicate a free propyl-SH chain [20,24]. The detection of the resonances at 40 and 21.3 ppm suggests the formation of disulfide bonds that can be created only in the case of the close proximity of the MPTMS molecules grafted onto the graphene sheets. Kao et al. [24] proposed that –S-S– bond formation is favored by the reactive sites of graphene oxide, similarly to that found with carbon nanotubes (CNT) [25].

Finally in the case of the Ga-M sample, in addition to the Ga peaks, metacryloxy resonances could be distinguished at 7.1 (C-1), 16.2 (C-7), 21.1 (C-2), 135.1 and 125.4 (C-5 and C-6, sharp peaks overlapped with the aromatic band), and 166.9 ppm (C-4), respectively, whereas C-3 was hidden by the C-OH Ga resonance. The chemical shift of C-4 proved that the metacryloxy ending group was preserved [26].

The reduced ability toward the condensation of MPTMS was confirmed by the ^29^Si CPMAS spectrum of Ga-M (Figure S4), which is characterized by a large number of T^2^ units (−58 ppm). T^3^ units (−66 ppm) increased from Ga-M to Ga-S and finally to Ga-N, in accordance with the increase in the condensation degree (Table S1).

**Figure 3:**
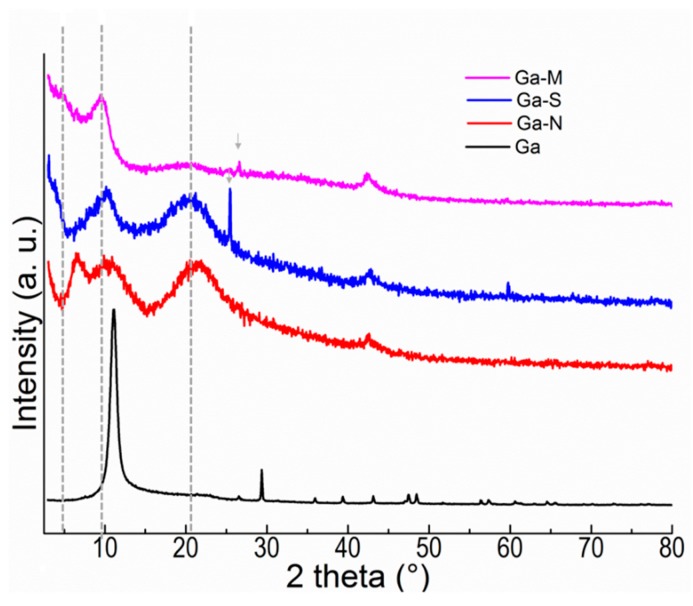
XRD diffractograms of the pristine and functionalized Ga samples.

Both pure and functionalized Ga samples were analyzed using the XRD technique (Figure 3) in order to confirm previous findings on functionalized GO [12] and correlate them with the NMR results. The XRD pattern of the starting Ga powders, obtained by drying the commercial aqueous solution, was characterized by an intense 001 basal peak located at 2θ = 11.16° (d = 0.792 nm); its correlation length, evaluated from Scherrer’s equation, is 10.2 nm. According to Lerf et al. [27], d-spacing values of about 0.79 nm can be attributed to a fully hydrated graphite oxide structure, with a monomolecular layer of water intercalated between the graphene oxide sheets. Moreover, from the evaluated correlation length, it is possible to estimate the number of stacked graphene oxide sheets oriented along the perpendicular direction to the diffracting (001) plane [28]; in this case, the value is around 12–13 layers. By comparing the XRD patterns of the functionalized samples with the Ga spectrum, the decrease in the intensity of the basal peak of graphene oxide was detected for the functionalized samples; the presence of the siloxane lattice was evidenced by the broad peak at about 2θ = 10° and the band at about 20–22°, both typical of the amorphous silsesquioxane structure [29]. In the Ga-N pattern, a signal at 6.43° (d = 1.37 nm) was clearly visible; analogously to the NMR study, the Ga:APTMS = 1:1 sample was analyzed (Figure S5) to strengthen the assignment of this peak to the graphene oxide counterpart. In Ga-N, the 001 peak shifted toward lower angles in relation with the increase of the interplanar distance d. The decrease in intensity and the shift of the basal peak of graphene oxide, together with the signal broadening evidenced by an increase in the FWHM value (from 0.79° in Ga to 1.35° in Ga-N), point out the presence of a strong interaction between silane amino groups and graphene oxide sheets, leading to a loss of the long-range order along the perpendicular direction to the diffracting plane; in fact, for the Ga-N sample, a stack of 4–5 sheets could be evaluated.

The progressive increase in the structural disorder of graphene oxide sheets can also be found for the Ga-S and Ga-M samples; in fact, the basal peak changed in a less intense shoulder located at around 3.5° for both samples. This effect can be related to the increase in the interplanar distances among the sheets, together with the loss of long-range order in the z-direction, as revealed by FWHM values higher than that of the Ga-N sample. At the same time, Figure 3 highlights the shift toward low angles of the siloxane band with the trend Ga-N (d = 0.810 nm) > Ga-S (d = 0.883 nm) > Ga-M (d = 0.910 nm), which may be due to the different length and arrangement of the silane organic chains. Additionally, the lineshape of the siloxane peak changed, the highest FWHM value (6.4°) was displayed by the Ga-N sample, attributable to a higher degree of interaction with GO sheets, while Ga-M (FWHM = 2.15°) showed the minimum interaction. Another common feature to arise from the analysis of the spectra was that all the other peaks present in the Ga spectrum vanished after the functionalization, except for a small peak located at d = 0.210–0.213 nm, due to the (100) direction of graphene oxide; this effect could be attributed to the maintenance of the structural order along the direction parallel to the diffracting planes [30]. Nevertheless, from the correlation lengths evaluated from the FWHM values, a lowering of the domain dimensions was recorded from Ga (>30 nm) to Ga-N (3.2 nm) and Ga-S (3.4 nm); again, the scarce interaction in the Ga-M sample also led to a higher degree of order in this direction, with an evaluated correlation length of 7.0 nm.

Finally, the presence of a small peak at 2θ = 26.6° in the Ga-M spectrum, attributable to the presence of graphite, could be interpreted as a consequence of a partial reduction reaction occurring in the graphene oxide layers as well as in the Ga-S sample that presented a very narrow signal at 25.5°.

**Figure 4:**
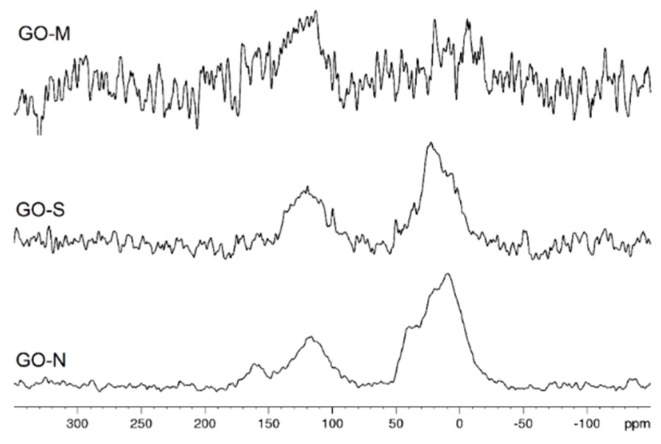
^13^C CPMAS spectra of samples GO-N, GO-S, and GO-M.

The experimental approach used to study the silanized Ga samples was also applied to the functionalized graphene oxide samples (GO-N, GO-S, and GO-M) prepared from the nitric acid etching of G powders [12]. Figure 4 shows the ^13^C CPMAS spectra of the samples GO-N, GO-S, and GO-M. The spectrum of GO (not reported) was flat due to the conductive character of the GO, which interferes with the polarization transfer of the experiment.

Theoretically, the CPMAS experiment permitted us to obtain good quality spectra for the organic component of the silane. Unfortunately, the low quantity of the grafted silane combined with the effect of the conductive graphene layers also caused both large widening and low S/N ratio of the peaks for the functionalized GO samples. However, it is worth noting that the broad resonance of graphene aromatic carbons, which is usually not detectable with CP experiments, can be observed in the spectra of Figure 3. This suggests that the functionalization allows partial cross-polarization between the protons of the silane propyl chain and the graphene neighboring C atoms.

Despite the low oxidation and functionalization degrees of the GO powders obtained by nitric acid etching with respect to Ga, the GO-N sample showed similarities to the Ga-N one. The band in the range of 50–0 ppm was given by the convolution of three peaks and there was also a weak C=O signal around 160 ppm, which has been previously assigned to amide bond formation. The GO-S spectrum presents similar features, but with a large decrease in the S/N ratio. For the GO-M sample, on the other hand, it is impossible to observe the peaks previously described, which suggests that the interaction between the silane and GO is very limited.

These results are comparable with those of the Graphenea series samples, as shown in Figure 1b, thus proving the suitability of the approach selected for the structural characterization of GO-organosilane interactions. Moreover, the conclusions of the NMR study on GO functionalized samples were in agreement with the evidence from both the FTIR and XRD study on the same samples reported in a previous paper [12]. Despite the low quality of the recorded XRD patterns and the graphitic structure of the GO samples prepared by nitric acid etching, the functionalization with the organosilanes led the graphitic and silsesquioxane counterparts to interact differently in relation with the different organic groups linked to silicon [12].

It is worth recalling that the properties of the composite layers based on acrylic resin loaded with 1% silanized GO powders made via cataphoresis were found to depend on the organosilane nature [12]. The trend in improving the layers’ corrosion resistance was in the order GO-N > GO-S > GO-M, which can be directly related with both the grafting ability and effect on the GO structural order of the different organosilanes evaluated through NMR and XRD.

Finally, another important feature of materials like the graphene oxide powders studied in this work is the presence of defects, which can significantly affect their chemical-physical properties. Most of the defects are related to the presence of unpaired electron spins, thus paramagnetic. Accordingly, they could be easily evaluated through ESR spectroscopy.

**Figure 5:**
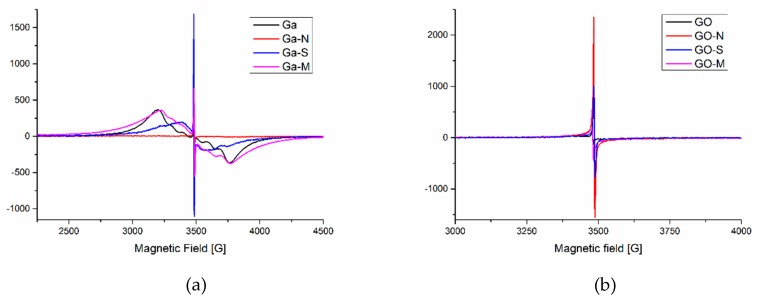
First derivative X-band cwESR spectra of the pristine and functionalized (**a**) Ga and (**b**) GO samples at room temperature.

Figure 5a,b show the ESR spectra of Ga, GO, and the corresponding functionalized samples. The ESR spectrum of Ga (Figure 5a, black) shows both a broad signal with a sextet hyperfine pattern and a small narrow peak (F) with a g value close to 2. The former signal, typical of diluted Mn^2+^ impurities (S = 5/2, I = 5/2) in polycrystalline samples, appears most likely as a consequence of the oxidation process with KMnO_4_ [31,32]. The latter is due to unpaired spins in both the C-related dangling bond and oxygen-based functional groups, such as carboxylic, alcoholic, epoxy and hydroxyl groups, whose presence has been already proved through ^13^C solid state NMR. It should be noticed that the presence of variable amounts of Mn^2+^ ions can affect the conductivity properties of the flakes as well as modify the barrier effect exerted by the GO fillers in polymeric matrices, leading to non-reproducible results.

The GO spectrum (Figure 5b, black) is characterized by a single sharp line centered at the g-value of 2.004. Since the starting graphene material (G) shows a relevant amount of C-based defects, according to the small broad peak with a g-value close to 2 and a high g-value signal probably due to the presence of spurious metals (Figure S6), it is likely that oxidation with nitric acid remarkably reduces the number of paramagnetic defects (vacancies, dangling bonds). Therefore, GO samples, partially oxidized without using KMnO_4_, offer better guarantees of conductivity and reproducibility with respect to the sample Ga in the preparation of the cataphoretic coatings [33].

The Ga functionalization with APTMS (Ga-N, Figure 5a) significantly reduced both the Mn impurities and the number of dangling bonds (C-related defects), whereas after reaction with MaPTMS (Ga-M) and, overall, MPTMS (Ga-S) the spectra displayed residual amounts of Mn^2+^ and the sharp signal due to the carbon radicals.

The spectra recorded on the GO functionalized samples (Figure 5b) showed only the sharp peaks related to the free radical species with a g-value of about 2.003–2.002.

Table 3 shows the intensity values of the sharp peak (F) attributed to defects/free electrons for a direct comparison of the sample properties, whereas the total area takes into account all the ESR signals, thus including all the other possible sources of spin density such as paramagnetic and metallic impurities, represented in the G and Ga-series by the broad ESR component. Interestingly, both Ga and GO functionalized samples showed the same trend with an increasing presence of defects from Ga-N to Ga-S to Ga-M and from GO-N to GO-S to GO-M. These results, therefore, strengthen the already observed similarity between the two series of samples and the possibility of generalizing the conclusions drawn for the model sample.

It can be noticed that the functionalization with the APTMS of both commercial Ga and GO strongly reduces the spin density. As reported above, functionalization with APTMS also reduced the metallic impurities of Ga. This is in agreement with the reported use of APTMS as fast and effective metal scavengers [34], thanks to the presence of the amino group.

According to [35], it is interesting to compare the ESR signal area that reflects all the unpaired electron spins with the area of the sp^2^ carbons in the ^13^C MAS NMR spectrum that could be related only to the unpaired spins coupled to ^13^C. Thomas et al. [35] found a correlation between the two types of data in their produced graphene oxide samples. In the present case, effective functionalization (samples Ga-S and Ga-N) seemed to reduce both the whole amount of unpaired spins (Table 3 column 3) and the ratio among the oxidized-C and sp^2^ C (Table 2, column 3).

## 4. Conclusions

The present paper describes the investigation of both the type and extent of interactions between a model commercial graphene oxide and three trialkoxysilanes bearing a different end group of the organic tail. The results of the Ga samples were compared with the ones obtained on GO prepared by nitric acid etching and subjected to the same functionalization, which was previously employed as a filler in protective coatings prepared by cataphoresis [12]. This approach allows for both the generalization of the results obtained on a model sample and for a relationship to be established among the structural and functional features of the GO-polymer nanocomposite layers for corrosion protection.

The multinuclear NMR study of the functionalized Ga samples highlights that the epoxy group is the preferential anchoring site for silane condensation, through its opening upon reaction with the Si–OH groups. The reaction with (X-propyl)trimethoxysilanes with amino-, mercapto-, and methacryloxy-X end groups, respectively, leads to a functionalization degree in the order –NH_2_ > −SH >> −O_2_CCCH_2_CH_3_. The methacryloxy function seems to hinder the grafting, whereas both aminopropyl and mercaptopropyl chains let the silane fill the space between the graphene sheets, most probably substituting the water molecules present in the pristine Ga, according to the XRD results. Moreover, the carbon chemical shifts of the mercaptopropyl chain indicate the formation of S–S bonds. Finally, according to the ^13^C and ^15^N results, the APTMS amino groups directly react with the edge carboxylic groups, leading to the formation of amide bonds. This evidence clearly explains the better performance imparted by the GO filler functionalized with APTMS to the acrylic layers deposited by cataphoresis onto metal surfaces [12]. Moreover, ESR spectroscopy proved that the presence of amino groups is beneficial for the removal of Mn impurities in commercial GO, acting as a metal scavenger and reducing the number of defects.

## Figures and Tables

**Table 1:** Assignment and amount of the main identified peaks [15,16] calculated from the profile fitting of the ^13^C MAS spectrum of Ga (Figure S1, Electronic Supplementary Materials).

δ(iso) ppm	Functional Group	Amount %
190	C=O	4.4
180	C=O	0.9
164	O=C–O	2.0
129	C=C	30.2
95	O–C–O (lactol)	3.0
69	C–OH	30.6
58	C–O–C (epoxy)	28.9

**Table 2:** Comparison of the number of oxidized C sites, extent of functionalization, and preferential anchoring sites in the different samples. Data were obtained through the profile fitting analysis of the ^13^C MAS spectra.

Sample	(C–OH + C–O–C(epoxy) + C=O) Normalized Area ^1^ (70–60, 180 ppm)	[COH + COC (epoxy)]/(R’O)_3_-Si-R Ratio ^2^	COH/COC (epoxy) Ratio
**Ga**	2.1		1.1
**Ga-N**	1.3	1.8	1.8
**Ga-S**	1.0	2.3	1.3
**Ga-M**	1.8	10 ^3^	1.0

^1^ Normalized with respect to the aromatic peak in the range of 134–125. ^2^ Ratio between COH + COC integrals and the integration of the region at 40–0 ppm, containing the three propyl methylene groups belonging to silane divided by three. According to propagation error theory, the reported value is a lower limit. ^3^ For sample Ga-M, the integration was done in the region 25–0 ppm, containing three peaks belonging to MaPTMS and the resulted integral is divided by three; this amount is subtracted from the 70–60 resonance that also convolutes the C-3 of the MaPTMS for comparison with the Ga-N and Ga-S results.

**Table 3:** ESR results of the modified graphene samples.

Sample	I_F_ (peak-to-peak) ^1^	Total Area (*10^6^) ^2^
G	7362.1	630
GO	275.8	4.69
GO-N	389.9	0.22
GO-S	1775.7	1.16
GO-M	9927.3	5.54
Ga	136.1	30.6
Ga-N	506.6	2.57
Ga-S	2790.6	19.6
Ga-M	1216.5	55.6

^1^ I_F_ is the intensity of the sharp peak. ^2^ The total area refers to the overall spectrum integral.

## Supplementary Materials

The following are available online at https://www.mdpi.com/1996-1944/12/23/3828/s1, Figure S1: Profile fitting analysis of Ga ^13^C MAS spectrum; Figure S2: FTIR spectra of samples Ga, Ga-N, Ga-S, and Ga-M; Figure S3: ^13^C CPMAS NMR spectrum of the sample Ga-N 1:1; Figure S4: ^29^Si CPMAS spectra of the samples Ga-N, Ga-S, and Ga-M; Figure S5: XRD spectrum of the Ga:APTMS 1:1 sample; Figure S6: First derivative cwESR spectrum of the graphene sample G; Table S1: Semi-quantitative profile fitting of T resonances based on CPMAS spectra.
