# Supplementary material for: Effect of the Organic Functional Group on the Grafting Ability of Trialkoxysilanes onto Graphene Oxide: A Combined NMR, XRD, and ESR Study
Source: Materials (Basel). 2019 Nov 21;12(23):3828. doi: 10.3390/ma12233828 (PMC6926944; doi:10.3390/ma12233828)
Supplement: Supplementary file 1 [file materials-12-03828-s001.pdf]

materials

Supplementary Materials

Effect of the Organic Functional Group on the Grafting Ability of Trialkoxysilanes onto Graphene Oxide: A Combined NMR, XRD, and ESR Study

Massimo Calovi [1], Emanuela Callone [2,*], Riccardo Ceccato [1], Flavio Deflorian [1], Stefano Rossi [1] and Sandra Dirè [2,*]

[1] Department of Industrial Engineering, University of Trento, 38123 Trento, Italy.; massimo.calovi@unitn.it (M.C.); riccardo.ceccato@unitn.it (R.C.); flavio.deflorian@unitn.it (F.D.); stefano.rossi@unitn.it (F.R.)
[2] "Klaus Müller" Magnetic Resonance Laboratory, Department of Industrial Engineering, University of Trento, 38123 Trento, Italy
* Correspondence: sandra.dire@unitn.it (S.D.); emanuela.callone@unitn.it (E.C.); Tel.: +39-04-6428-2463 (E.C.); +390461282456 (S.D.)

Received: 31 October 2019; Accepted: 19 November 2019; Published: date

Profile fitting analysis of the ^{13}CNMR spectra

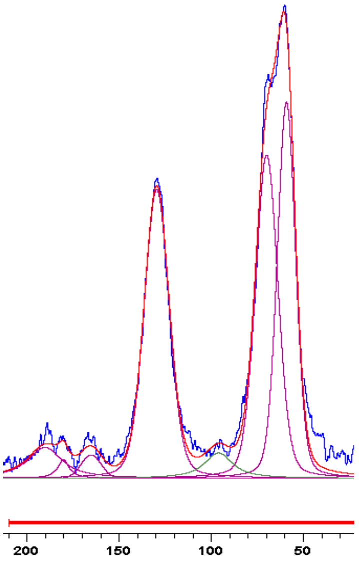

Figure S1. Profile fitting of the ^{13}C MAS NMR spectrum of sample Ga.

As an example, Figure S1 shows the result of the fitting procedure of the ^{13}C MAS NMR spectrum of sample Ga (Figure 1a) undertaken by using the devoted Bruker Topspin3.6 software routine. It provides the position, linewidth (FWHM), and area of the different components through an iteration procedure that was stopped when an acceptable confidence level was obtained (90%) without constrains on the three parameters.

FTIR study of the functionalized Ga samples

The FTIR spectra were collected on KBr pellets in transmission mode (64 scans, 4 cm-1 resolution) using a Varian 4100 instrument.

The FTIR spectra of the pristine and functionalized Ga samples are shown in Figure S2. The Ga sample presents the typical features of oxidized graphene [12], with bands at 1713 and 1238 cm^{-1} attributed to the reactive oxidized groups. Comparing the spectra of Ga-N and Ga, the presence of the intense band in the range 1200–1000 cm^{-1}, associated with SiO asymmetric stretching vibrations,

confirmed the effective silane grafting on graphene sheets. The siloxane band appeared less visible in Ga-S and particularly in Ga-M, whose FTIR spectrum was dominated by graphene spectral features Therefore, in agreement with the NMR results, the FTIR spectra confirmed that the Ga-N sample had the greatest functionalization degree and Ga-M had the lowest one. The functionalization with APTMS, MPTMS, and MaPTMS of the GO samples produced by nitric acid etching of graphene [12] was also studied by FTIR, and a similar trend was observed despite the low oxidation degree of GO.

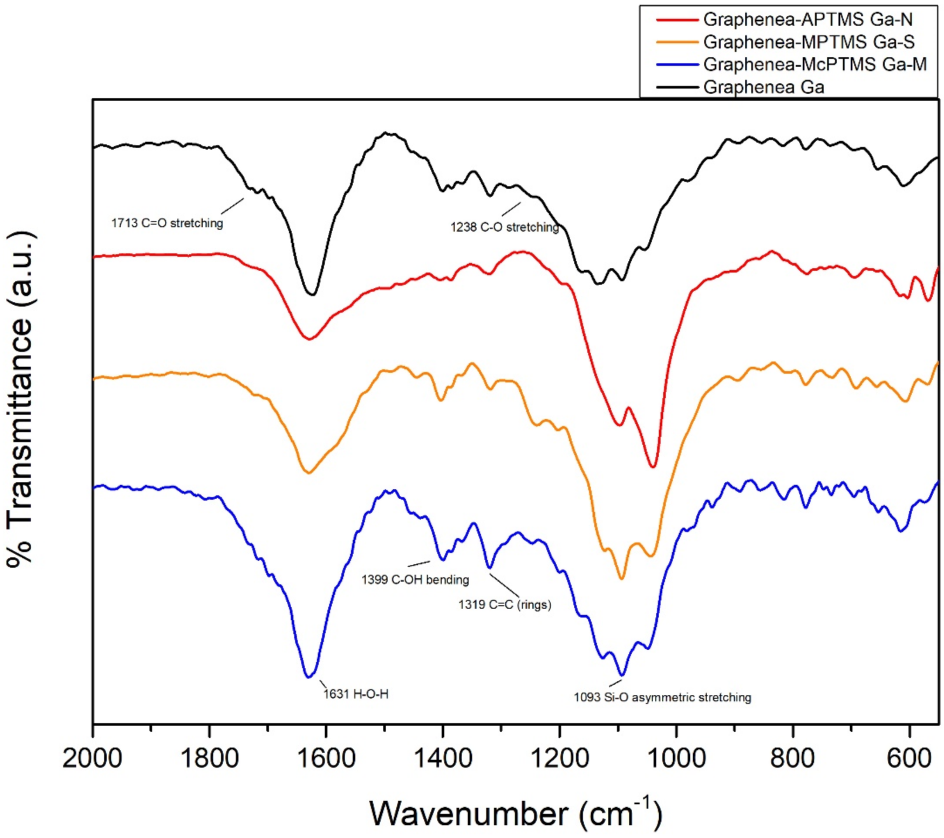

Figure S2. The FTIR spectra of samples Ga, Ga-N, Ga-S, and Ga-M.

[13.] C CPMAS analysis of the sample prepared with Ga:APTMS 1:1 ratio

The ^{13}C CPMAS spectrum of the sample prepared with the Ga:APTMS 1:1 ratio (Figure S3) clearly showed the signals of the methylene carbons of the propyl chain. The Cβ resonance splits as observed in Ga-N, but with a remarkable difference in the intensity of the downfield component that appeared increased. Moreover, the intensity of the signal at 164 ppm also increased with respect to the Ga-N spectrum (Figure 1b). The graphene signals could not be detected anymore as a consequence of the large amount of organosilane employed.

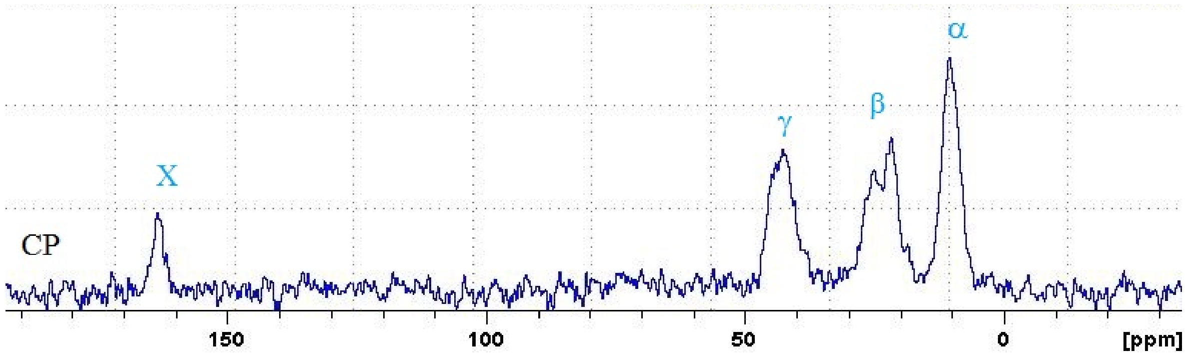

Figure S3. ^{13}C CPMAS NMR spectrum of the sample Ga-N 1:1.

^{29}Si CPMAS analysis of functionalized Ga samples

^{29}Si CPMAS experiments were also run in order to evaluate the inorganic counterpart of the functionalizing agents. ^{13}C NMR has already shown that the silanes were fully hydrolyzed, according to the absence of the –OMe peak, with the exception of Ga-M, and condensed (according to the chemical shift of Cα). The spectra reported in Figure S4 present two partially overlapped peaks belonging to T^3 at −66 ppm and T^2 units at −58 ppm, respectively. The former represents the Si–O–Si units (i.e., the fully condensed units), indicating the amount of bulk organosilane. The latter represents both the Si–OH terminal units and Si–O–$C_{graphene}$ grafted units. From a qualitative point of view, the sample Ga-M presents the highest amount of T^2 units among all of the samples (Table S1). This can be explained by the lower condensation ability of MaPTMS, with respect to APTMS or MPTMS. A further consideration can be made by taking into account that the intensity of the T^2 units due to Si–OH is favored by the cross polarization effect, since the protons are closer to Si centers than in Si–OC or Si–OSi. Thus, the lower amount of T_2 in Ga-S and Ga-N could also be ascribed to a majority of Si–O–C bonds, where the C source can be only the Ga. This seems particularly true for Ga-N because the lineshape of the T^2 peak was even broader, suggesting the presence of multiple components.

Table S1. Semi-quantitative profile fitting of T resonances based on CPMAS spectra.

	**%T^2**	**%T^3**
Ga-M	64.7	35.3
Ga-S	39.3	60.7
Ga-N	29.1	70.9

Finally, the S/N ratio gives a qualitative idea of the amount of silica-based materials that remains within the graphene oxide sheets. Thus, the amount of grafted MaPTMS is much lower with respect to APTMS and MPTMS, in agreement with the FTIR and ^{13}C NMR results.

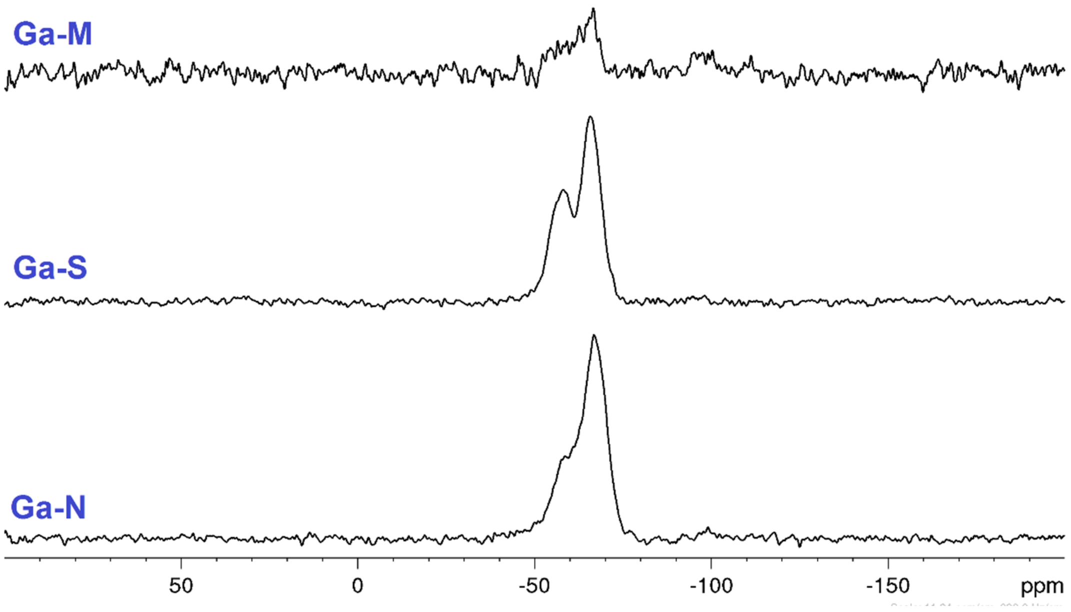

Figure S4. ^{29}Si CPMAS spectra of the samples Ga-N, Ga-S, and Ga-M.

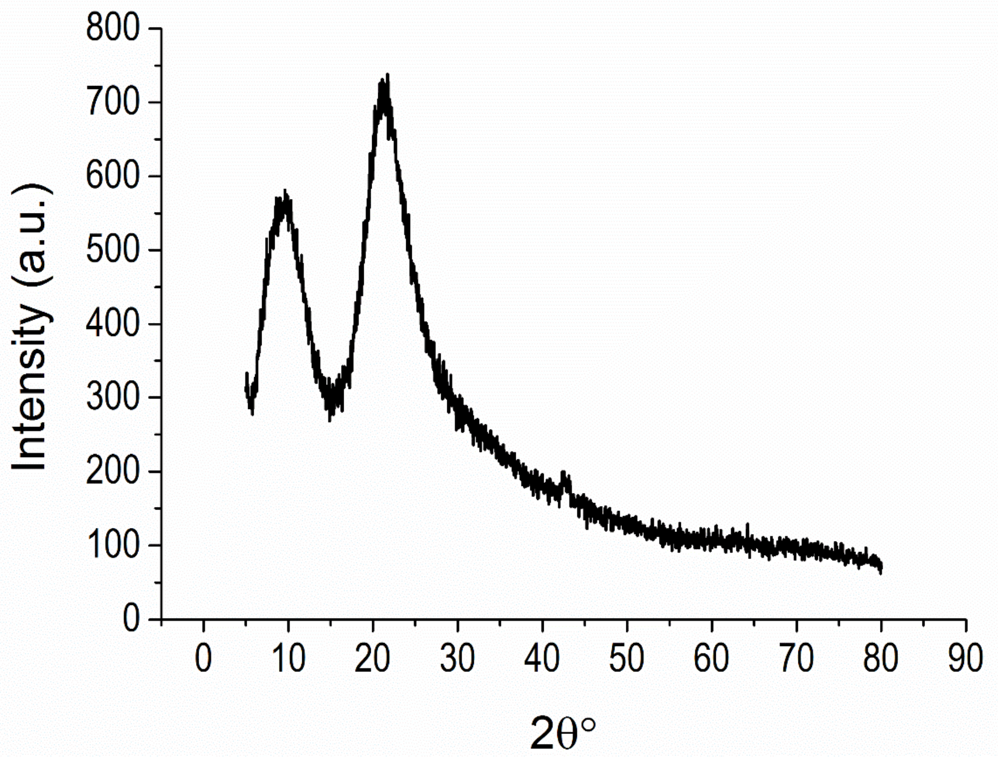

Figure S5. XRD spectrum of the Ga:APTMS 1:1 sample.

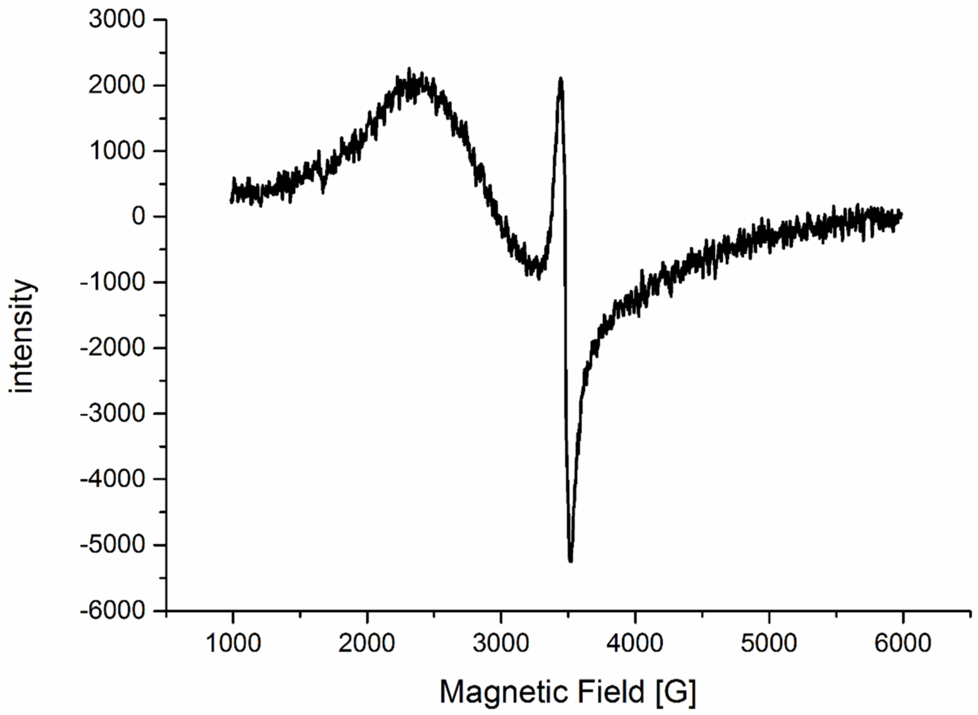

Figure S6. First derivative of the cwESR spectrum of graphene sample G.
